# Nondestructive Phenomic Tools for the Prediction of Heat and Drought Tolerance at Anthesis in* Brassica* Species

**DOI:** 10.34133/2019/3264872

**Published:** 2019-05-22

**Authors:** Sheng Chen, Yiming Guo, Xavier Sirault, Katia Stefanova, Renu Saradadevi, Neil C. Turner, Matthew N. Nelson, Robert T. Furbank, Kadambot H. M. Siddique, Wallace A. Cowling

**Affiliations:** ^1^The UWA Institute of Agriculture, The University of Western Australia, Perth, WA 6001, Australia; ^2^UWA School of Agriculture and Environment, The University of Western Australia, Perth, WA 6001, Australia; ^3^High Resolution Plant Phenomics Centre, Australian Plant Phenomics Facility, CSIRO Agriculture and Food, Canberra, ACT 2601, Australia; ^4^ARC Centre of Excellence for Translational Photosynthesis, Australian National University, Canberra, ACT 2601, Australia

## Abstract

Oilseed Brassica species are vulnerable to heat and drought stress, especially in the early reproductive stage. We evaluated plant imaging of whole plant and flower tissue, leaf stomatal conductance, leaf and bud temperature, photochemical reflectance index, quantum yield of photosynthesis, and leaf gas exchange for their suitability to detect tolerance to heat (H) and/or drought (D) stress treatments in 12* Brassica* genotypes (G). A replicated factorial experiment was set up with 7 d of stress treatment from the beginning of anthesis with various levels of three factors* H*,* D*, and *G*. Most phenomics tools detected plant stress as indicated by significant main effects of* H*,* D*, and* H×D*. Whole plant volume was highly correlated with fresh weight changes, suggesting that whole plant imaging may be a useful surrogate for fresh weight in future studies. Vc*max*, the maximum carboxylation rate of photosynthesis, increased rapidly on day 1 in H and H+D treatments, and there were significant interactions of* G×H* and* G×D*. Vc*max* of genotypes on day 1 in H and H+D treatments was positively correlated with their harvested seed yield. Vc*max* on day 1 and day 3 were clustered with seed yield in H and H+D treatments as shown in the heatmaps of genotypic correlations. TPU, the rate of triose phosphate use, also showed significant positive genotypic correlations with seed yield in H+D treatments. Flower volume showed significant interactions of* G×H* and* G×D* on day 7, and flower volume of genotypes on day 7 in H was positively correlated with their harvested seed yield. There were few interactions of* G×H* or* G×D* for leaf stomatal conductance, leaf and bud temperature, photochemical reflectance index, and quantum yield of photosynthesis. Vc*max*, TPU, and volume of flowers are potential nondestructive phenomic traits for heat or combined heat and drought stress tolerance screening in* Brassica* germplasm.

## 1. Introduction

Heat stress and water deficit often occur in the field simultaneously and have deleterious effects on crop growth, development, and productivity worldwide [1, 2]. A 30% reduction in gross primary productivity across Europe in 2003, for example, was estimated to be due to heat and drought [3]. Damage to US agriculture caused by a combination of heat and prolonged drought (≥ US$120 billion) was severalfold higher than that caused by drought alone (≤ US$20 billion) between 1980 and 2004 [4] and is predicted to reduce US agricultural output by up to 4.3% per year from 2010 to 2050 [5]. Heat and drought stress accompanying global climate change are the likely cause of a recent plateau in crop yields in Australia [6].

Oilseed* Brassica napus* (oilseed rape, canola) is an important crop traditionally grown in high-rainfall areas but is vulnerable to heat and drought stress especially during the early reproductive stage. It has a relatively narrow gene pool [7] and this situation is accentuated in Australia due to 3 decades of closed recurrent selection [8].* B. rapa* (field mustard, turnip), a diploid ancestor of tetraploid* B. napus*, is a potential source of diversity for genetic improvement in* B. napus* [9].* B. rapa* is distributed widely on a global scale with the centre of origin in the Old World and centres of diversity in Asia [10–14], with some types flourishing in heat and drought-affected regions [15]. Genotypic variation for heat and drought resistance has been reported recently in* B. rapa* [16, 17], and a tolerant genotype responded to simulated drought with rapid expression of gene networks for general stress responses and programmed cell death [18].

In* B. napus*, temperatures greater than 29.5°C during flowering resulted in seed yield losses in Ottawa, Canada, and seed yield decreased as heat stress increased [19]. Young et al. [20] showed that a temperature of 35°C for 4 h each day for 1 or 2 weeks after the initiation of flowering in* B. napus *reduced fruit and seed development, pollen germination, and* in vivo* pollen tube growth. In contrast, Annisa et al. [16] found that pollen viability remained above 87% in all accessions under heat stress with and without water deficits during early flowering in six spring-type* B. rapa *accessions, but seeds failed to develop at high temperatures as a result of inhibition of fertilization or postfertilization processes. Bud number and length and pod number produced under heat stress might provide a useful preliminary screen for heat stress tolerance [16]. Heat stress imposed during flowering negatively impacted photosynthetic capacity and grain yield in* B. napus* [21].

High-throughput genotypic profiling has been greatly achieved in recent decades, but it has not been matched by fast and accurate crop phenotyping methods, and thus reliable plant phenotyping under various environments has become a major bottleneck for crop genetics and breeding [22–24]. Nondestructive imaging such as visible and near-infrared reflectance techniques has been developed for diagnosing plant physiological and stress status [25, 26]. Thermal imaging and canopy temperature are sensitive measures of the stomatal response to abiotic stress [27, 28]. Infrared imaging on leaves has previously been used to screen wheat and sorghum populations for stress resistance [29]. Image-based plant phenomics has been successfully applied to phenotype the whole plant response to nitrogen and phosphorous nutrition [30], to dissect the genetic architecture of temporal salinity responses in rice [31], to reveal salinity tolerance loci [32], and to study genetic variation in 245 diverse chickpea accessions for salinity tolerance [33]. Floral bud temperature was a useful indicator of water status in the reproductive organs of* B. rapa* [34]. Chlorophyll fluorescence was used as a surrogate for photosynthesis and photosynthetic damage arising from heat and drought stress [35–37]. Richards [38] found that chlorophyll stability was related to yield in* B. napus* in response to drought stress, and chlorophyll fluorescence was used to predict drought tolerance in durum wheat [39]. Rapid chlorophyll loss during a 3-day heat treatment was linked to heat susceptibility and reduced grain filling in wheat [40]. Digital biovolume, a high-throughput phenotyping measure based on imaging techniques in the RGB domain, was successfully applied in durum wheat and tomato to identify genotypes resilient to water stress and to discriminate biostimulant treatments [41]. Therefore, plant phenomics provides high-throughput, nondestructive phenotyping tools that could potentially be applied to large-scale screening for tolerance to drought and heat stress in crops.

In this study, we measured the response of 12 oilseed* Brassica* genotypes to heat and drought stress at the early flowering stage using a range of nondestructive plant phenomics tools, such as whole plant imaging, leaf stomatal conductance, leaf and bud temperature, photochemical reflectance index, quantum yield of photosynthesis, and leaf gas exchange. The objectives of this study were to test (1) which plant phenomics tools detected drought and/or heat stress in oilseed* Brassica*; (2) which plant phenomics tools showed differential effects among* Brassica* genotypes in their response to drought and/or heat stress; and (3) which nondestructive traits were associated with biomass or grain yield of genotypes and hence could be used to predict the drought and/or heat tolerance of oilseed* Brassica*.

## 2. Materials and Methods

### 2.1. Plant Materials

Twelve* Brassica* genotypes, including five* B. rapa*, five* B. napus*, and two* B. juncea *genotypes, were chosen based on wide genetic diversity and potential heat and drought tolerance phenotypes from previous studies (Table 1). The five* B. rapa* genotypes represented the spectrum of genetic diversity and geo-distribution in* B. rapa* [12, 14] and included one heat-tolerant* B. rapa* genotype (accession ATC95217) [16]. The five* B. napus* genotypes were Australian breeding lines or cultivars with different level of tolerance and sensitivity to drought and/or heat stress as investigated previously [42]. The two* B. juncea* genotypes included one heat-tolerant genotype (accession ATC95209) [16].

### 2.2. Plant Management

Experiments were conducted at the controlled environmental facility of the High Resolution Plant Phenomics Centre (HRPPC) in Canberra, Australia. Seed was sown in four batches at two-weekly intervals. The first batch was used as a pilot study to fine-tune the protocol for phenomic tools and to test and adjust the environment. We measured the available soil water content in pots, the rate of water loss in the combined heat and drought treatments, and the amount of water supply needed to manage the drought treatment and maintain well-watered conditions in the heat and control treatments. The remaining three batches were used as biological replicates in the experimental design.

At each time of sowing, five 8.1 L pots 230 mm in depth (standard P250 pots, Garden City Plastics, Australia) were prepared for each genotype. In each pot there was 4.5 kg of canola potting mix, which consisted of 50% fine composted pine bark, 20% coco peat and 30% brown river sand plus 1.0 g of gypsum per kg with its final pH at ~6.0. Five seeds were sown at a depth of 10 mm in each pot. Germination occurred in a growth chamber at 15°C constant temperature. After 12 days, pots were transferred to a cold room (2°C) for 4 weeks of vernalization to condense the flowering variation between genotypes (and individual plants within genotypes) to less than one week. At the end of vernalization, all pots were transferred to a glasshouse, in which the maximum and minimum temperatures were set at 25°C (at midday) and 8°C (at night) with an average of 15°C, ensuring that there was neither heat stress nor frost stress on the plants prior to the imposition of treatments at first open flower. The seedlings were thinned to two healthy and strong plantlets in each pot. Plants were watered daily in the glasshouse. The photoperiod for the plants in this experiment in both the growth chambers and glasshouse was kept constant at 16 h light (06.00-22.00 h) and 8 h dark. HORTICO Aquasol™ (a fast-acting soluble fertilizer with trace elements, 23:4:18 N:P:K) was applied fortnightly until flowering. Disease and/or pest control followed HRPPC's regulation and chemicals were applied when required.

### 2.3. Drought and Heat Stress Treatments during Anthesis

One day before the first open flower, the soil in each pot was saturated with water and 140 g of high-density polyethylene white beads (Qenos Pty Ltd, Victoria, Australia) was applied to the soil surface to minimize soil evaporation. At 08.00 h on the day when the first open flower was seen on the main stem of a plant, the pot weight including the soil and the growing plant was measured and moved into the controlled environment growth cabinets to commence the 7-d treatments during anthesis. In this way, each plant received the relevant treatment at the same development stage. The growth cabinets were maintained at 400 *μ*mol mol^−1^ CO_2_ and 60% relative humidity, with light intensity 600 *μ*mol m^−2^ s^−1^ of PAR at plant height level for 16 h followed by 8 h of dark throughout the 7-d treatments.

Four different treatments were applied for 7 d during anthesis: (1) control (C), with normal temperature and normal moisture, that is, well-watered; (2) drought (D), with normal temperature and water stress; (3) heat (H), with high temperature and well-watered; and (4) combined heat and drought stress (H+D). The normal temperature treatment was set at 23°C day (06.00 - 22.00 h) and 15°C night (22.00 - 06.00 h). The H treatment had a constant temperature of 25°C at night for 8 h (22.00 - 06.00 h), and during the light period the temperature gradually increased to 35°C at 11.00 h, maintained for 4 h (11.00 - 15.00 h), and then gradually decreased to 25°C at 22.00 h [16] (Supp. Figure S1). These conditions are based on field experience in Australia, where daily maximum temperatures were recorded in late September 2017 (during anthesis in grain crops) in the grain-growing belt of Australia (https://www.timeanddate.com/weather/australia/dubbo/historic?month=9&year=2017) and are likely to occur more frequently in future [43].

Each pot was weighed to measure the water loss and soil water content (SWC) each day after treatment (DAT) began. We followed procedures developed previously [17, 34] to control drought stress in pots, so that severe drought stress was achieved in the D and H+D treatments before DAT7, and death due to drought was avoided. The water treatments were controlled by daily water supply in the early morning (08.00-09.00 h) as developed in the pilot study. For the well-watered treatment, 100% of the water lost in the last 24 h was supplied to each pot each DAT; for the water-stressed treatments, 50% of the water lost in the last 24 h was supplied to each pot each DAT.

In order to develop the method in preliminary experiments, we measured the minimum SWC after 7 d of stress treatment. In the combined H + D treatment SWC was 34.0% and in the D treatment it was 42.7%, while the minimum in C treatment was 78.9% and in the H treatment was 66.9%, which are not considered to be water-stressed conditions (Supp. Figure S2). These changes in SWC are similar to those we achieved in previous pot experiments for control and water-stressed treatments in* B. rapa* [17, 34].

### 2.4. In-Cabinet Rapid Nondestructive Measurements

At DAT1, DAT3, DAT5, and DAT7, all plants were subjected to a set of in-cabinet rapid nondestructive measurements between 11.00 and 13.00 h, including leaf stomatal conductance (LC), leaf and bud temperature, photochemical reflectance index (PRI), and photosystem II quantum yield of photosynthesis (Qy). The LC of the youngest fully expanded leaf was measured using an SC1 leaf porometer (Decagon Devices, Washington, USA). LC was recorded as the sum of the adaxial and abaxial conductance. The temperature of a newly opened floral bud and a nearby leaf was measured with Impac® Model IN 15 plus (LumaSense Technologies GmbH, Santa Clare, USA) infrared thermometer with a minimum 2.2 mm diameter measurement area. A separate digital thermometer with a 1 s response time was used to measure the ambient temperature. For buds, the vertically oriented buds just prior to open flower stage were chosen and measured with the device oriented horizontally from the side. For leaves, a horizontal portion of the first fully opened leaf was measured with the device oriented vertically from above. Leaf and bud temperatures and the ambient temperature were recorded simultaneously with four repeat measurements per leaf and bud. The temperature differences between bud and ambient environment (T1), leaf and ambient environment (T2), and bud and leaf (T3) were calculated. PRI was recorded using a PlantPen model PRI 200 (Photon Systems Instruments, Drásov, Czech Republic) chlorophyll meter for the estimation of leaf light-use efficiency and photosynthesis by measuring the relative chlorophyll content of the leaf* in situ*. Qy was recorded using Fluorpen FP100 (Photon Systems Instruments, Drásov, Czech Republic) for photosynthetic efficiency of photosystem II.

### 2.5. Leaf Gas Exchange

At DAT1, DAT3, and DAT7, the gas exchange of each genotype was measured with a LiCOR 6400XT (LiCOR Inc., Lincoln Nebraska) portable photosynthesis system with 20 × 30 mm head for gas exchange analysis. LiCor devices were set up in the normal and H temperature growth cabinets. Block temperature was maintained at either 23°C or 35°C to match the temperature conditions in each cabinet. The relative humidity was maintained at 60% in the chamber, the same as in both cabinets. Irradiance on the leaf was maintained at 600 *μ*mol m^−2^ s^−1^ with auto-programmed change in intercellular CO_2_ concentration at 0, 50, 75, 100, 150, 400, 800, and 1600 *μ*mol mol^−1^. All gas exchange readings were taken between 11.00 and 15.00 h to ensure peak conductance in the diurnal cycle of the plants as well as at the highest temperature in the H treatment. CO_2_ assimilation rate (A) was measured relative to increasing intercellular CO_2_ partial pressure (Ci) on the youngest fully expanded leaf.

A/Ci curves were constructed and fitted to a model [44] to reveal the following parameters: maximum carboxylation rate allowed by Rubisco (Vc*max*) from the Rubisco-limited curve; photosynthetic electron transport rate (ETR) from the ribulose 1,5-bisphosphate (RuBP) regeneration-limited curve; and the rate of triose phosphate use (TPU) from the TPU-limited photosynthesis curve [44].

### 2.6. Plant Growth Imaging

At DAT0, DAT3, and DAT7, all plants were subjected to plant growth imaging with a Scanalyzer (LemnaTec GmbH, Aachen, Germany). The imaging system uses two red-green-blue (RGB) cameras and produces a top view, a 0° side view, and a 90° rotated side view in each image capture (Figure S3). The three images for each plant were then analysed with the Scanalyser imaging software automated algorithm. Plant pixels are separated from nonplant pixels, to create a two-dimensional plant area for each image in pixels. This two-dimensional plant area was then calibrated and converted to mm^3^ to create a plant volume as follows:

(1) $$\begin{array}{c} \text{Plant volume}\left(mm^{3}\right)=\sqrt{Topview\left(mm^{2}\right)\times Sideview0^{{}^{\circ}}\left(mm^{2}\right)\times Sideview90^{{}^{\circ}}\left(mm^{2}\right)} \end{array}$$

Plant volume over time under different treatments was plotted for all genotypes and used in comparative analyses. Yellow flower volume was readily separated from total plant volume on the basis of pixel colour type.

### 2.7. Fresh Weight of Above-Ground Plants and Seed Yield

At the end of DAT7 (17.00 – 18.00 h), plants in the first and second biological replicates were cut at the soil level and the fresh weight (FW, g) measured, and plants in the third biological replicate were returned to the glasshouse and grown to maturity. All seed pods from each plant were harvested at maturity and dried at 32°C for 7 days. Seeds were threshed manually and cleaned by Vacuum Separator (Kimseed, WA Australia). The oven-dry seed yield (SY, g), seed number counted and 100-seed weight (SW, g) from each genotype were measured.

### 2.8. Statistical Analysis

A factorial experiment was designed with three factors (genotype, heat, and drought), with two levels of heat (normal and high temperature), two levels of drought (normal and water-stressed), 12 genotypes in each treatment, and three randomised complete blocks (replicates). Four environments (stress treatments) were imposed at anthesis: C, H, D, and H+D. The blocking structure was represented by three biological replicates sown two weeks apart. The genotypes were measured randomly in each growth cabinet at each DAT for each measurement, so that there was no need for additional modelling of a time-dependent correlation structure, typical for repeated measure experiments. A linear mixed model was used for all traits (response variables), where the main effects of* Drought *(*D*)*, Heat *(*H*)*, Genotype *(*G*), and their interactions were fitted as fixed effects and* Block* as a random effect. The measurements at DAT0, where available, were fitted as a covariate. A few of the traits exhibited nonhomogeneous variance, and logarithmic transformation was applied. The analyses were conducted using statistical software ASReml-R v3 [45] and R environment, R3.0.1 [46]. Genotypic correlations between all the phenotypic traits under each treatment were shown by heatmaps, which were produced using R package superheat (https://cran.r-project.org/web/packages/superheat).

## 3. Results

### 3.1. In-Cabinet Rapid Nondestructive Measurements

#### 3.1.1. Photochemical Reflectance Index (PRI) and Quantum Yield of Photosynthesis (Qy)

PRI and Qy showed a significant main effect of* G*, but few significant interactions of* G×H*,* G×D*, or* G×H×D* (Table 2), which shows that while genotypes differed in these traits, there was little change in ranking of genotypes across C, H, D, and H+D treatments. The mean values of PRI and Qy varied only slightly between treatments C, H, D, and H+D at DAT 1, 3, 5, 7 (Supp Table S1).

#### 3.1.2. Temperature of Floral Buds and Leaves

As with PRI and Qy, the temperature difference between ambient and floral buds (T1), between ambient and leaves (T2), and between floral bud and leaf temperature (T3) showed significant main effects of* G*, but few significant interactions of* G×H*,* G×D* or* G×H×D* (Table 2). While genotypes differed in T1, T2, and T3 under stress, there is little change in ranking of genotypes for these traits across C, H, D, and H+D treatments.

**Figure 1:**
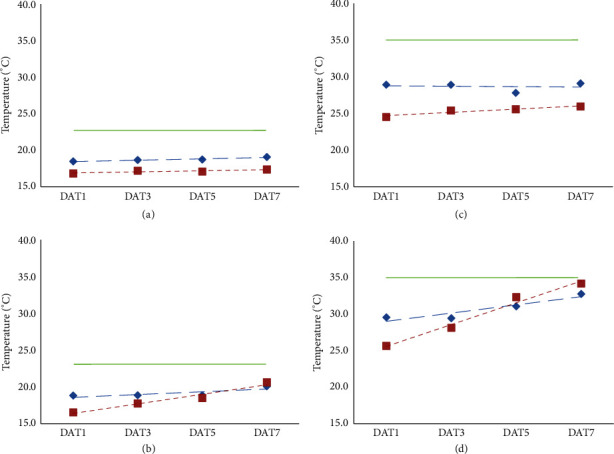
The temperature of newly opened buds (blue diamonds) and adjacent leaves (red squares) compared with ambient temperature (green line) from 1 (DAT1) to 7 days (DAT7) after exposure to drought, heat, and combined heat and drought treatments relative to the control. Data are means of the 12 genotypes. The fitted linear regressions are shown, with significance of the slope parameter for DAT: (a) Control: T*bud* = 18.37 + 0.09DAT;* p* = 0.174; T*leaf* = 16.82 - 0.02DAT,* p* = 0.861. (b) Drought: T*bud* = 18.41 + 0.19DAT,* p* = 0.023; T*leaf* = 15.78 + 0.47DAT,* p* < 0.001. (c) Heat: T*bud* = 28.82 - 0.03DAT,* p* = 0.174; T*leaf* = 24.35 + 0.27DAT,* p* = 0.086. (d) Combined: T*bud* = 28.29 + 0.63DAT,* p* < 0.001; T*leaf* = 24.09 + 0.91DAT,* p* < 0.001.

However, there was a significant* H×D* interaction for these traits (Figure 1, Table 2). In the C and H treatments, the floral bud temperature was consistently higher than the leaf from DAT1 to DAT7 and did not change significantly over time (Figures 1(a) and 1(c)). In the D treatment, the temperature of the floral bud was higher than the leaf at DAT1, but leaf temperature increased relative to bud temperature and was slightly higher than the floral buds at DAT7 (Figure 1(b)). In the H+D treatment, the temperature of floral buds and leaves both increased significantly during the stress treatment; however, the leaf temperature increased even more rapidly over time compared with bud temperature, much faster than in the D treatment alone (Figure 1(d)), and this results in the significant* H×D* interaction.

#### 3.1.3. Leaf Stomatal Conductance (LC)

**Figure 2:**
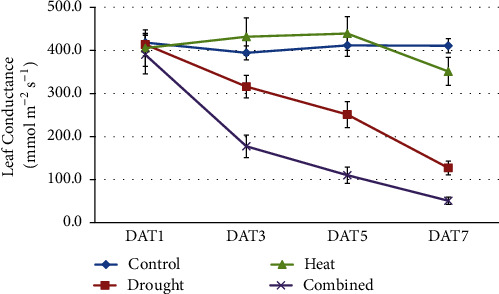
Leaf stomatal conductance from 1 (DAT1) to 7 days (DAT7) after exposure to drought, heat, and combined heat and drought treatments and the well-watered control treatment at normal temperature. Data are means of the 12 genotypes ± one standard error.

As with the above traits, for LC there was a significant main effect of* G*, but few significant* G×H*,* G×D*, or* G×H×D* interactions (Table 2). While genotypes differed in LC, there was little change in ranking of genotypes across C, H, D, and H+D treatments. However, the mean values of LC varied greatly between treatments C, H, D, and H+D at DAT3, DAT5, and DAT7 (Figure 2, Supp. Table S1). LC dropped quickly from DAT1 to DAT7 in the D and H+D treatments, as expected for drought stress, but did not change greatly in the H treatment which was well-watered (Figure 2) confirming that the effects of heat stress were not due to lack of water. However, H+D had a significantly greater effect on LC than D alone by DAT3 (Figure 2).

### 3.2. Photosynthetic Parameters Inferred from LiCor Gas Exchange Measurements

**Figure 3:**
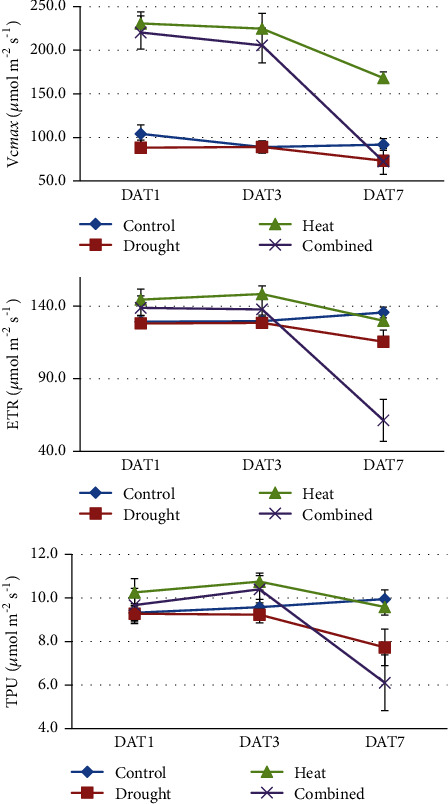
Photosynthetic parameters, maximum carboxylation rate (Vc*max*), photosynthetic electron transport rate (ETR), and the rate of triose phosphate use (TPU) as inferred from A/Ci curves (Sharkey et al., 2007) after 1, 3, and 7 days of treatment (DAT) of drought, heat, and combined heat and the well-watered control treatment at normal temperature. Data are means of the 12 genotypes ± one standard error.

The maximum carboxylation rate allowed by Rubisco (Vc*max*), the photosynthetic electron transport rate (ETR), and the rate of triose phosphate use (TPU), as inferred from the A/Ci curves, showed significant main effects of* G*,* H*, and *D* and* H×D* interaction at some DAT, and some significant* G×H*,* G×D*, or* G×H×D* interactions (Table 2). The behaviour of Vc*max *over time was particularly interesting (Figure 3). The Vc*max* in H and H+D treatments was more than double compared to the level in the C and D treatments at DAT1 and DAT3 (Figure 3, Supp. Table S1). The* G×H* and* G×D* interactions for Vc*max* at DAT1 were significant, which indicates that the* Brassica* genotypes changed ranking for Vc*max* in H and D treatments compared with C.

### 3.3. Plant Growth Imaging

**Figure 4:**
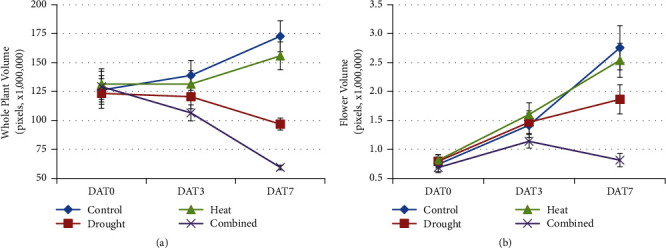
Whole plant (a) and yellow flower (b) volume after 1, 3, and 7 days of treatment (DAT) of drought, heat, and combined heat and the well-watered control treatment at normal temperature. Data are means of the 12 genotypes ± one standard error.

The volume of whole plant (VolWP) and volume of yellow flowers (VolF) showed significant main effects of* G*,* H*, and *D* and* H×D* interaction at some DAT, and some significant* G×H*,* G×D*, or* G×H×D* interactions (Table 2). VolWP decreased by 44% under D and 66% under H+D treatments by DAT7 (Figure 4(a)).

VolF increased in C and H treatments from DAT0 to DAT7 (Figure 4(b)). However, VolF at DAT7 in the D and H+D treatments was 32% and 70% lower than the control, respectively (Figure 4(b)). This reflects a significant* H×D* interaction for VolF at DAT3 and DAT7 (Table 2). Importantly, there were significant* G×H* and* G×D* interactions for VolF at DAT7 (Table 2), and therefore VolF at DAT7 should be explored further as a potentially useful indicator of H and D tolerance.

### 3.4. Fresh Weight and Seed Yield under Drought and Heat Stress

The fresh weight (FW) at DAT7 and seed yield (SY) at maturity showed significant main effects of* G*,* H*, and* D*, and FW shows significant* G×H*,* G×D*, and* G×H×D* interactions (Table 2). As shown in Supp. Table S1, the average FW per plant of the 12* Brassica* genotypes in the control treatment was 427.9 g (range 108.5 to 791.1 g) and average SY per plant in the control treatment was 3.0 g (range 0.8 to 7.7 g). FW declined by 48.2%, 11.4%, and 69.4% in D, H, and H+D treatments, respectively. SY declined by 40.8%, 57.3%, and 57.3% in the D, H, and H+D treatments, respectively. The average 100-seed weight (SW) of the 12* Brassica* genotypes varied significantly across genotypes (Table 2) (range 0.20 to 0.52 g) but was not affected by treatments (Table 2, Supp. Table S1).

### 3.5. Genotypic Correlations

#### 3.5.1. Fresh Weight vs. Phenomics Traits

**Figure 5:**
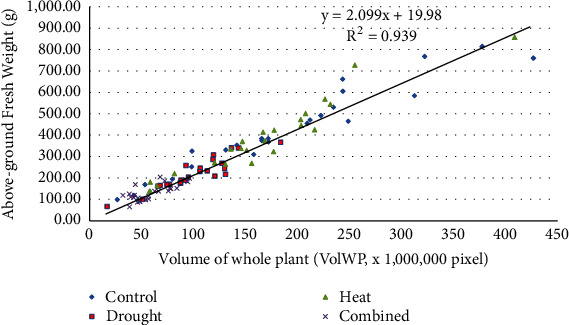
The regression of the whole plant volume (VolWP) with the above-ground fresh weight of 12* Brassica* genotypes 7 days after exposure to no stress (control), drought, heat, and combined heat and drought treatments. One fitted linear regression is given across all treatments. Individual plant values for VolWP and fresh weight are shown.

**Figure 6:**
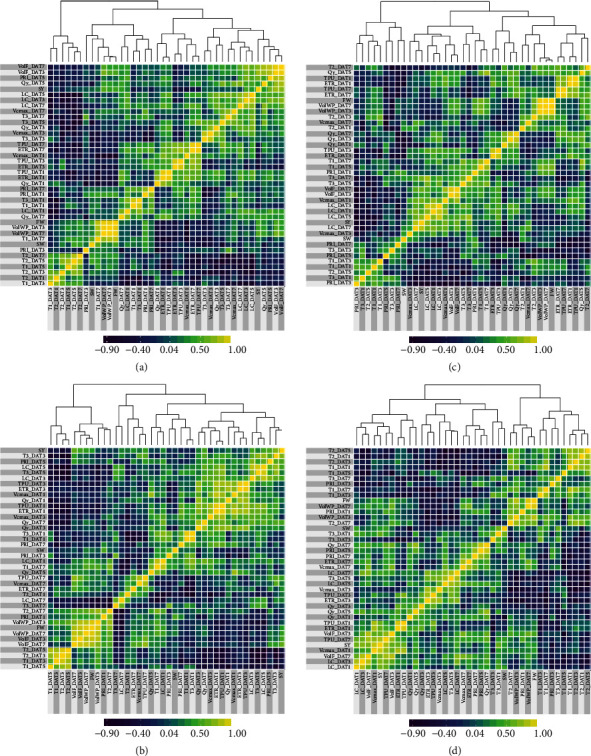
Heatmaps of genotypic correlations for various phenomic and agronomic traits at various days after treatment (DAT) measured in control (a), drought (b), heat (c), and combined heat and drought (d) stress treatments. For trait abbreviations see Table 2.

FW was strongly positively correlated with VolWP of genotypes at DAT3 and DAT7 at DAT7 in all treatments (Table 3). VolWP was an excellent surrogate for FW at DAT7 across all genotypes and treatments (Figure 5). Genotypic correlations for FW and VolWP_DAT7 and VolWP_DAT3 were always clustered together under C, D, H, and H+D conditions (Figure 6).

Under C, D, and H+D treatments, genotypes with higher values of T2 (the temperature of the leaf below ambient) tended to have higher FW; however, the correlation between FW and T2 under any treatment was not strong (*r* < 0.5) (Table 3).

PRI, Vc*max*, ETR, and TPU at DAT1, DAT3, DAT5, or DAT7 showed no significant genotypic correlations with FW under any stress conditions (Table 3).

#### 3.5.2. Seed Yield vs. Phenomics Traits

**Figure 7:**
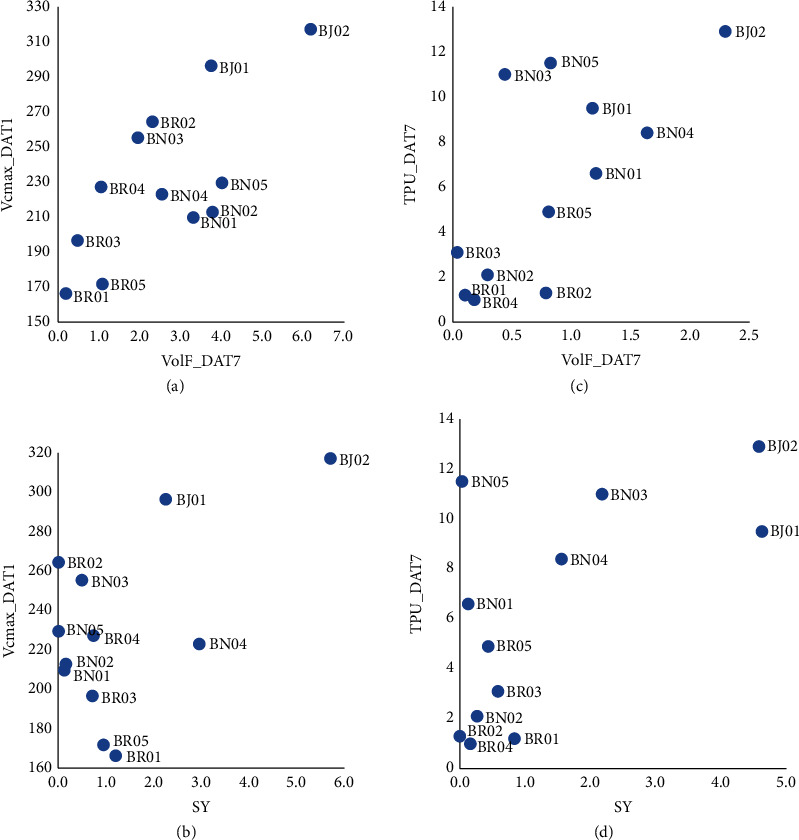
Genotypic response for Vc*max*_DAT1 vs VolF_DAT7 (a) and Vc*max*_DAT1 vs SY (b) under heat stress and for TPU_DAT7 vs VolF_DAT7 (c) and TPU_DAT7 vs SY (d) under combined heat and drought stress. For trait abbreviations see Table 2, and for genotype abbreviations see Table 1.

SY showed significant positive genotypic correlations with Vc*max* at DAT1 (Vc*max*_DAT1) in the H and H+D treatments (Table 4); that is, the higher the maximum carboxylation rate of genotypes under these stresses at DAT1, the higher the SY. BJ02, the genotype with the highest SY under H had the highest Vc*max*_DAT1 (Figure 7(b)). A similar result was observed for TPU_DAT7 in the H+D treatment, that is, the higher the rate of triose phosphate use at DAT7, the higher the SY (Table 4 and Figure 7(d)). This is consistent with the heat maps of genotypic correlations, which show that SY and Vc*max*_DAT1 and Vc*max*_DAT3 were clustered together in H and H+D treatments (Figures 6(c) and 6(d)), but not in C or D treatments (Figures 6(a) and 6(b)). Likewise, TPU_DAT7 was clustered together with SY in H+D, but not in C, H, or D alone (Figure 6).

There was also a significant positive genotypic correlation between flower volume at DAT7 (VolF_DAT7) and SY, but only in the H treatment (Tables 4 and 5). That is, the higher the VolF_DAT7 in the H treatment, the higher the SY of those genotypes. The genotypes with the highest SY (BJ01, BJ02, and BN04) under H also had the highest VolF_DAT7 under H.

LC, PRI, and Qy showed no significant genotypic correlations (*p* ≤ 0.05) with SY at DAT1, DAT3, DAT5, or DAT7 under any stress conditions. There was a significant negative genotypic correlation for T2 (leaf temperature relative to ambient) at DAT7 and SY in the H treatment, and a positive genotypic correlation between T1 (bud temperature relative to ambient) and SY at DAT1 in H (Table 4); that is, genotypes with higher bud temperature at DAT1 under H treatment tended to have higher SY. However, the* G×H* and* G×D* interactions for T1 and T2 were not significant (Table 2), indicating that there was no change of ranking of genotypes for T1 and T2 in H, D, or H+D treatments compared with C.

#### 3.5.3. Volume of Flowers vs. Phenomics Traits

VolF_DAT7 was highly correlated with other plant-volume-related traits, such as VolF_DAT3 and VolWP_DAT3. However, genotypic correlations between FW and VolF (VolF_DAT3 and VolF_DAT7) were clustered together only under D, and not under C, H, or H+D conditions (Table 5, Figure 6). Genotypic correlations also existed between VolWP_DAT7 and the three photosynthesis-related traits, i.e., Vc*max*_DAT1 under H, ETR_DAT7 under D, and TPU_DAT7 under D and H+D (Table 5). There were positive genotypic correlations between Vc*max*_DAT1 and TPU_DAT7 and VolF_DAT7, and genotype BJ02 had the highest levels in each case (Figures 7(a) and 7(c)).

VolF_DAT7 was also correlated with T2_DAT7 and T3_DAT7 under D, LC_DAT5 under H and LC_DAT3 and LC_DAT7 under H+D (Table 5).

## 4. Discussion

### 4.1. Drought and Heat Treatments

We evaluated the impact of heat and drought treatments for 7 days during anthesis, which is a critical period for impact of stress on plant growth and final grain yield of canola and other* Brassica* species [16, 17, 19, 34]. The heat and drought stress treatments in this research caused significant changes in several phenomics traits after 7 days of stress during anthesis, which ultimately impacted on FW and SY, as shown by significant main effects of heat and drought for these traits in the analysis of variance (Table 2).

In previous growth chamber experiments with drought during anthesis, we showed that the predawn leaf water potential of* Brassica* plants in small pots was closely correlated with SWC during the drought treatment, and drought stress was evident after 2-3 days of treatment when SWC fell below 60% and LC fell from 400 to less than 150 mmol m^−2^ s^−1^, at which point leaf water potential was less than -1 MPa [34]. In those experiments, LC was significantly inhibited by drought as was final SY [17, 34]. In the current experiments, SWC fell below 60% in both D and H+D treatments by DAT5 (Supp Figure S2), and LC was less than 150 mmol m^−2^ s^−1^ in both D and H+D treatments by DAT7 (Figure 2). Also, SY was significantly reduced in the D and H treatments compared with C (Table 2, Supp Table S1). The D treatment resulted in a gradual increase in drought stress during the seven-day treatment (Supp. Figure S2), although drought stress was not evident on DAT1, when LC was 400 mmol m^−2^ s^−1^ in all treatments (Figure 2).

Importantly, we avoided drought stress in the heat treatments and vice versa. The SWC was consistently kept above 70% in the H treatment and close to 80% SWC in the C treatment (Supp. Figure S2). The LC in H was above 400 mmol m^−2^ s^−1^ from DAT1 to DAT5, the same level as in C (Figure 2 and Supp. Table S1). In many previous studies, especially those based in the field [19], heat effects may be confounded by drought effects. In this study, the two treatments were not confounded, and therefore we have valid estimations of the main effects of* H*,* D*, and *G* and the interactions* G×H*,* G×D*, and* G×H×D*.

### 4.2. Interactions of* G×H*,* G×D*, and* G×H×D*

Significant interactions of* G×H*,* G×D*, and* G×H×D* (Table 2) for various traits indicate that genotypes changed ranking under heat and drought stress for that trait. Several nondestructive phenomics traits (Vc*max*, ETR, TPU, VolWP, and VolF) showed changes in genotype ranking under stress (Table 2), and in some cases the genotypic values under stress were correlated with changes in tissue volume or fresh weight of genotypes and/or with final seed yield (Figure 6). The most promising of these was Vc*max*_DAT1 and TPU_DAT7 which showed significant genotypic correlations with VolF_DAT7 and SY (Figure 7). We conclude that these phenomics traits have potential for further development as nondestructive indicators of heat and drought stress tolerance in Brassica species, following validation in a broader range of genotypes. In contrast, we found no evidence of significant* G×H*,* G×D*, and* G×H×D* for LC, T1, T2, T3, PRI, and Qy (Table 2).

### 4.3. Floral Bud and Leaf Temperature and LC Are Strong Indicators of Stress Status and Potential Reproductive Failure

Canopy temperature has been proposed as a rapid selection tool for abiotic stress tolerance among crop genotypes [34, 47, 48]. In our experiments, leaf and floral bud temperature increased over 7 days of drought stress, but not in the control or heat stress treatment. LC also decreased under drought stress but not under control or heat stress. The increase in leaf temperature over time is presumably because of a reduced rate of transpiration in both D and H+D treatments due to drought stress, which was not present in the C and H treatments. This confirms, as in our previous experiments on heat stress tolerance in* B. rapa* [16], that drought stress was avoided in the high temperature treatment.

In our previous research, a 10-day transient water stress during reproductive development in* B. rapa* increased bud temperature and resulted in reproductive failure including both flower abortion and pod abortion and thus elevated floral bud temperature over time was potentially a useful indicator of drought stress in reproductive organs [34]. Also, seeds of* B. rapa* failed to develop in pods under high temperatures when there was no drought stress [16]. This is in agreement with studies in* B. napus* which show that heat stress during flowering reduces micro- and mega-gametophyte fertility, induces fruit abortion, and disrupts seed production in* B. napus* [20]. In groundnuts, the flower number was quantitatively related to floral bud temperatures during the day over the range 28–43 °C [49]. Therefore, high floral bud temperature is commonly associated with reproductive failure in plants.

Our results for T3 (the temperature difference between the floral bud and adjacent leaf) agree with Guo et al. [34] that temperature increases slower in floral buds than in leaves under drought stress. T3 also changed from negative to positive over time, and this was associated with a higher rate of water loss (or stomatal conductance) in floral buds. Whether this is because the water status of the bud is higher than the leaves or because the stomata remain open at lower water potentials in buds than in leaves is not known.

Nevertheless, there were few genotypic correlations between T1, T2, or T3 with SY or FW under drought (Table 2). We conclude that T1, T2, and T3 are not promising phenomics traits for drought tolerance in Brassica species.

### 4.4. Photosynthetic-Related Plant Phenomic Traits

Vc*max *is derived from the first state of the A/Ci curve and indicates the maximum carboxylation rate of Rubisco, i.e., the Rubisco catalytic capacity, which is a key parameter in assessing photosynthetic assimilation rate [50]. The higher the Vc*max*, the higher the potential efficiency of photosynthesis and the greater the rate of carboxylation.

In this study, there were significant interactions of* G×H* and* G×D* for Vc*max* at DAT1 (Table 2). Vc*max* remained at the basal level under C or D treatments but rapidly increased to a high level under H or H+D stress at DAT1 and DAT3 (Figure 3). The genotypic correlations in Figure 6(c) (H treatment) show that Vc*max*_DAT1 and Vc*max*_DAT3 are clustered together with SY, that is, genotypes with higher Vc*max* at DAT1 or DAT3 tend to have higher SY at harvest (e.g., genotype BJ02; Figure 7(b)). This is preliminary evidence that Vc*max* after 1 or 3 days of H at anthesis may be a useful phenomics trait to predict heat stress tolerance in Brassica. More work is required to evaluate the significant interaction of* G×D* at DAT1 (Table 2) which was not validated by heat maps where Vc*max* was not associated with SY in the D treatment (Figure 6).

Measurement of the initial slope of the A/Ci response (Vc*max*) is used to screen for Rubisco activase limitations. In sweet potato, spinach, and tobacco, there is no evidence of limitations in Rubisco activase at high temperature [37]. In spinach leaves, Vc*max* increases from low to high temperatures without evidence of limitation at high temperatures [51]. In tobacco, photosynthesis was limited by Vc*max* above 32°C (whilst by ETR below 32°C) [52]. This is consistent with our results where Vc*max* in H and H+D was more than double that in C and D treatments at DAT1, whereas there was little change in TPU and ETR at DAT1 in H and H+D (Figure 3).

ETR, derived from the second state of the A/Ci curve, is related to RuBP regeneration. When ETR is high, photosynthesis is very efficient through the greater ability for RuBP regeneration. ETR in the light reaction of photosystems I and II is crucial in the ATP synthesis and NADP^+^ reduction required to energize the processes of the Calvin cycle. TPU, the third phase of photosynthesis, reflects the point at which carbohydrates and sugars are generated for plant growth, thus being the final measure of photosynthetic efficiency. In this study, both ETR and TPU decreased under combined heat and drought stress, and there was a significant change in ranking of genotypes for ETR with heat and TPU with drought. Therefore, ETR and TPU might be useful for relative tolerance of* Brassica* genotypes to heat and/or drought stress.

While the three photosynthetic parameters are quite promising in our experiment, it is worth pointing out that the LiCOR 6400XT photosynthesis system is portable but relatively heavy, and that measuring A–Ci curves to estimate Vc*max* and ETR is a time-consuming and laborious process. A possible alternative is hyperspectral reflectance (350–2500 nm), which has been shown to be a useful surrogate for a range of photosynthetic traits including Vc*max* and ETR on intact wheat leaves in the glasshouse and under field conditions, with correlation coefficients up to 0.62 for Vc*max* and 0.70 for ETR [53]. This hyperspectral screen in wheat takes only 20 s per leaf. Such a rapid hyperspectral screen would substantially support the use of Vc*max* and ETR as phenomic traits for large-scale drought/heat tolerance screening in* Brassica* once the suitability of this measurement was proven in* Brassica*.

### 4.5. Image-Based Plant Phenomic Traits

Digital biovolume based on imaging techniques has been useful to identify genotypes of durum wheat and tomato resilient to water stress [41]. In this study, the LemaTec Scanalyser was used to image plant growth during 7 days of drought and/or heat stress at anthesis, and the whole plant volume (VolWP) was highly correlated with the fresh weight of plants. The significant regression between whole plant volume and fresh weight suggests that whole plant imaging may be a useful surrogate for fresh weight in future studies. The volume of yellow flowers (VolF_DAT7) was correlated with seed yield of genotypes under heat stress and thus should be further explored as a nondestructive surrogate for heat and drought stress tolerance in oilseed* Brassica* crops.

Future research on the role of plant imaging as a phenomics tool for* Brassica* heat and drought stress tolerance should include measurements of pod number, pod size, and number of seeds per pod. Plant imaging may provide a useful prediction of flower or pod abortion, which frequently results from stresses occurring at early reproductive stages [16, 17, 20, 54].

## 5. Conclusions

In our experiments, several phenomics traits show potential to discriminate between Brassica genotypes for heat and drought stress tolerance. The main trait of interest is Vc*max*, and genotypes with high Vc*max*_DAT1 and Vc*max*_DAT3 under heat stress were the highest yielding genotypes. TPU_DAT7 is also positively and significantly correlated with seed yield under combined heat and drought stress. Therefore, Vc*max* and TPU are two putative phenomics parameters for tolerance to heat and combined heat and drought stress in* Brassica*. More work is necessary across a wider range of genotypes to confirm these interesting results, and to evaluate faster methods such as hyperspectral reflectance [53], which may act as a surrogate for Vc*max* and permit screening of hundreds of progeny for heat tolerance.

Plant growth imaging also has potential in phenomics studies of heat and drought stress tolerance in* Brassica*. The flower volume of genotypes on day 7 of heat treatment was positively correlated with their final seed yield. Whole plant volume on day 7 in all treatments was highly correlated with fresh weight on day 7, suggesting that whole plant imaging may be a useful surrogate for fresh weight in future studies.

## Figures and Tables

**Table 1:** Origins of the twelve *Brassica* genotypes used in this study.

Genotype	Species	Accession	Country of Origin	Seed Provider^a^	Note	Ref
BJ01	*B. juncea*	ATC95209	Pakistan	ATFCC	Landrace	[16]
BJ02	*B. juncea*	JR049	Australia	UoM	Breeding line	[42]
BN01	*B. napus*	AV-Garnet	Australia	UoM	Cultivar released in 2009	[42]
BN02	*B. napus*	Monty-028DH	Australia	CBWA	Doubled haploid from cultivar Monty	[42]
BN03	*B. napus*	RT117	Australia	UoM	Breeding line	[42]
BN04	*B. napus*	Tanami	Australia	CBWA	Cultivar released in 2007	[42]
BN05	*B. napus*	Tarcoola	Australia	UoM	Cultivar released in 2009	[42]
BR01	*B. rapa*	ATC92037	India	ATFCC	Landrace	[12, 14]
BR02	*B. rapa*	Fuding Baicai	China	NPZ	Landrace	[12, 14]
BR03	*B. rapa*	111	India	NPZ	Landrace yellow sarson	[12, 14]
BR04	*B. rapa*	ATC92240	India	ATFCC	Landrace	[12, 14]
BR05	*B. rapa*	ATC95217	Indonesia	ATFCC	Landrace	[16]

^a^UoM: The University of Melbourne; CBWA: Canola Breeders Western Australia Pty Ltd; ATFCC: Australian Temperate Field Crops Centre; NPZ: Norddeutsche Pflanzenzucht Hans-Georg Lembke KG, Germany.

**Table 2:** The mean squares (MS) and degrees of freedom (df) of treatments and residuals for genotype (G), heat (*H*), drought (*D*), and their interactions^a^ for 11 phenomic traits^b^ and 3 agronomic traits^c^ as measured on 12 *Brassica* genotypes from 1 to 7 days after treatments (DAT) during anthesis. The statistical significance of main effects and interactions is shown as 0.01 < *p* < 0.05 (*∗*) and *p* < 0.01 (*∗∗*). “ns” and “NA” indicate “not significant” and “not applicable,” respectively.

Trait	DAT	Source of variation														Residuals	
		*G*		*H*		*D*		*G×H*		*G×D*		*H×D*		*G×H×D*			
		df =11		df =1		df =1		df =11		df =11		df =1		df =11		df	MS
*(i) Six in-cabinet rapid phenotyping traits*																	

PRI	1	0.0040	*∗∗*	0.0006	*∗*		ns		ns		ns		ns		ns	96	0.0001
	3	0.0028	*∗*	0.0007	*∗*		ns		ns		ns		ns		ns	96	0.0001
	5	0.1000	*∗*		ns		ns	0.1093	*∗*		ns		ns		ns	96	0.0049
	7		ns		ns	0.0005	*∗*		ns		ns		ns		ns	96	0.0001

Qy	1	0.0260	*∗∗*	0.0060	*∗*		ns		ns		ns		ns		ns	96	0.0010
	3	0.0419	*∗∗*		ns		ns		ns		ns		ns		ns	96	0.0007
	5	0.0318	*∗∗*		ns	0.0069	*∗∗*		ns		ns		ns		ns	96	0.0008
	7	0.0409	*∗∗*	0.0156	*∗∗*	0.0330	*∗∗*		ns		ns	0.0336	*∗∗*		ns	96	0.0014

T1	1		ns	71.700	*∗∗*		ns		ns		ns		ns		ns	96	5.6368
	3	118.452	*∗∗*	89.508	*∗∗*		ns		ns		ns		ns		ns	96	3.3334
	5	122.400	*∗*	67.800	*∗∗*	97.300	*∗∗*		ns		ns	86.500	*∗∗*		ns	96	5.3684
	7	123.480	*∗∗*		ns	193.590	*∗∗*		ns		ns	61.460	*∗∗*		ns	96	4.7786

T2	1	132.360	*∗*	462.590	*∗∗*		ns		ns		ns		ns		ns	96	5.9198
	3	179.160	*∗∗*	264.880	*∗∗*	96.86	*∗∗*		ns		ns	42.140	*∗∗*		ns	96	5.1332
	5	284.740	*∗∗*		ns	599.43	*∗∗*		ns		ns	255.470	*∗∗*		ns	96	8.1280
	7	203.710	*∗∗*	31.920	*∗*	1187.95	*∗∗*		ns		ns	219.530	*∗∗*		ns	96	6.3080

T3	1	207.403	*∗∗*	169.743	*∗∗*		ns		ns		ns		ns		ns	96	6.7461
	3		ns	33.046	*∗∗*	55.406	*∗∗*		ns		ns	29.348	*∗*		ns	96	4.6506
	5	101.137	*∗*		ns	209.639	*∗∗*		ns		ns	51.073	*∗∗*	100.395	*∗*	96	4.9548
	7		ns		ns	418.200	*∗∗*		ns		ns	55.600	*∗∗*		ns	96	5.2742

LC	1	447132	*∗∗*		ns		ns		ns		ns		ns		ns	96	7670.82
	3	440562	*∗∗*		ns	211692	*∗∗*		ns		ns	91991	*∗∗*		ns	96	4992.83
	5	263299	*∗∗*	28906	*∗*	539613	*∗∗*		ns		ns	63756	*∗∗*		ns	96	5936.44
	7		ns	18381	*∗*	651370	*∗∗*		ns		ns		ns		ns	96	3676.20

*(ii) Three photosynthetic traits derived from A/Ci curve*																	

Vcmax	1	38159	*∗∗*	201158	*∗∗*	2100	*∗∗*	35625	*∗∗*	17665	*∗∗*		ns		NA	12	293.98
	3		ns	191108	*∗∗*		ns		ns		ns		ns		NA	12	2407.81
	7		ns	17042	*∗∗*	39130	*∗∗*		ns		ns	17887	*∗∗*		NA	12	1018.22

ETR	1	16189	*∗∗*	2031	*∗∗*		ns	4424	*∗∗*		ns		ns		NA	12	114.25
	3	8118	*∗∗*	2337	*∗∗*		ns		ns		ns		ns		NA	12	285.85
	7		ns	10776	*∗∗*	23629	*∗∗*		ns		ns	7068	*∗∗*		NA	12	886.04

TPU	1	117.500	*∗∗*		ns		ns		ns		ns		ns		NA	12	1.9685
	3	53.700	*∗∗*	16.300	*∗∗*		ns		ns		ns		ns		NA	12	1.5817
	7	153.900	*∗∗*		ns	96.100	*∗∗*		ns	125.300	*∗∗*		ns		NA	12	3.6774

*(iii) Two plant growth imaging-derived phenotyping traits*																	

VolWP	3	463877	*∗∗*		ns	19157	*∗∗*		ns	31012	*∗∗*		ns		ns	96	1245.17
	7	261725	*∗∗*	30020	*∗∗*	267973	*∗∗*		ns	126866	*∗∗*		ns		ns	96	1461.15

VolF	3	102.935	*∗∗*		ns		ns	20.590	*∗∗*		ns	3.112	*∗*		ns	96	0.5380
	7	241.570	*∗∗*	18.740	*∗∗*	65.870	*∗∗*	34.950	*∗∗*	54.320	*∗∗*	5.680	*∗*		ns	96	1.1831

*(iv) Three agronomic traits*																	

FW	7	101256	*∗∗*	116824	*∗∗*	1238679	*∗∗*	6001	*∗∗*	41512	*∗∗*	10410	*∗*	5545	*∗∗*	48	1883.69

SY	At harvest	12.789	*∗∗*	14.719	*∗∗*	4.490	*∗*		ns		ns		ns		NA	12	0.9399

SW	At harvest	0.0198	*∗∗*	0.0101	*∗*		ns		ns		ns		ns		NA	12	0.0015

^a^ *G*×*H*: Genotype by Heat interaction; *G*×*D*: Genotype by Drought interaction; *H*×*D*: Heat by Drought interaction; *G*×*H*×*D*: Genotype by Heat by Drought interaction.

^b^ The phenomic traits include (i) six in-cabinet rapid phenotyping traits: photochemical reflectance index (PRI), photosystem II quantum yield (Qy), leaf conductance (LC), and the temperature differences between (a) bud and ambient environment (T1), (b) leaf and ambient environment (T2), and (c) bud and leaf (T3); (ii) three photosynthetic traits derived from A/Ci curves: maximum carboxylation rate allowed by Rubisco (Vc*max*), photosynthetic electron transport rate (ETR) and the rate of triose phosphate use (TPU); and (iii) two plant growth imaging derived phenotyping traits: whole plant volume (VolWP) and flower volume (VolF).

^c^ The agronomic traits include fresh weight (FW) of the whole plant at DAT7, and seed yield (SY) and 100-seed weight (SW) of each plant at maturity.

**Table 3:** Correlations between fresh weight at 7 days after treatment (DAT7) and phenomic traits^a^ across 12 *Brassica* genotypes under nonstress (control), drought, heat and combined heat, and drought stress conditions. Traits without significant correlations (*p* ≥ 0.05) are not shown.

Trait	Control		Drought		Heat		Combined	
Qy_DAT3					-0.421	*∗*		
T1_DAT1					0.472	*∗*		
T1_DAT5					0.515	*∗∗*		
T1_DAT7	0.519	*∗∗*						
T2_DAT5							0.421	*∗*
T2_DAT7	0.444	*∗*	0.490	*∗*			0.480	*∗*
T3_DAT5					0.429	*∗*	-0.414	*∗*
LC_DAT7							-0.471	*∗*
VolWP_DAT3	0.944	*∗∗*	0.889	*∗∗*	0.928	*∗∗*	0.795	*∗∗*
VolWP_DAT7	0.958	*∗∗*	0.914	*∗∗*	0.960	*∗∗*	0.716	*∗∗*
VolF_DAT3	0.608	*∗∗*	0.764	*∗∗*			0.532	*∗∗*
VolF_DAT7	0.473	*∗*	0.810	*∗∗*	0.574	*∗∗*		

^a^ Phenomic traits are indicated as phenomics parameter at different days after treatment (DAT); phenomics parameters include (i) six in-cabinet rapid phenotyping traits: photochemical reflectance index (PRI), photosystem II quantum yield (Qy), leaf conductance (LC), and temperature difference between (a) bud and ambient environment (T1), (b) leaf and ambient environment (T2), and (c) bud and leaf (T3); (ii) three photosynthetic traits derived from A/Ci curves: maximum carboxylation rate allowed by Rubisco (Vc*max*), photosynthetic electron transport rate (ETR), and the rate of triose phosphate use (TPU); and (iii) two plant growth imaging derived phenotyping traits: whole plant volume (VolWP) and flower volume (VolF).

^b^ Correlation (*r*) significance values are shown as *p* <0.05 (*∗*) and <0.01 (*∗∗*).

**Table 4:** Correlations between seed yield and phenomic traits^a^ across 12 *Brassica* genotypes under nonstress (control), drought, heat and combined heat, and drought stress conditions. Traits without significant correlations (*p* ≥ 0.05) are not shown.

Trait	Control		Drought		Heat		Combined	
PRI_DAT3	0.763	*∗*						
T1_DAT1					0.651	*∗*		
T2_DAT7					-0.707	*∗*		
Vc*max*_DAT1					0.670	*∗*	0.878	*∗∗*
TPU_DAT7							0.798	*∗∗*
VolF_DAT7					0.735	*∗*		

^a^ Phenomic traits are indicated as in Table 3.

^b^ The three agronomic traits are seed yield per plant (SY), 100-seed weight (SW), and fresh weight of above-ground plant at DAT7 (FW).

^c^ Correlation (*r*) significance values are shown as *p* <0.05 (*∗*) and <0.01 (*∗∗*).

**Table 5:** Correlations between flower volume after 7 days of treatments (VolF_DAT7) and phenomic^a^ and agronomic^b^ traits across 12 *Brassica* genotypes under nonstress (control), drought, heat and combined heat, and drought stress conditions. Traits without significant correlations (*p* ≥ 0.05) are not shown.

Trait	Control		Drought		Heat		Combined	
Qy_DAT5	0.493^c^	*∗∗*						
T2_DAT7			0.408	*∗*				
T3_DAT7			–0.426	*∗*				
LC_DAT3	0.459	*∗*					0.415	*∗*
LC_DAT5					0.413	*∗*		
LC_DAT7							0.470	*∗∗*
Vc*max*_DAT1					0.724	*∗*		
ETR_DAT7			0.797	*∗∗*				
TPU_DAT7			0.727	*∗*			0.774	*∗∗*
VolWP_DAT3			0.631	*∗∗*	0.469	*∗∗*	–0.075	
VolWP_DAT7	0.539	*∗∗*	0.808	*∗∗*	0.515	*∗∗*	0.397	*∗*
VolF_DAT3	0.903	*∗∗*	0.777	*∗∗*	0.800	*∗∗*	0.616	*∗∗*
SY					0.735	*∗*		
FW			0.662	*∗*				

^a^ Phenomic traits are indicated as in Table 3.

^b^ The three agronomic traits are seed yield per plant (SY), 100-seed weight (SW), and fresh weight of above-ground plant at DAT7 (FW).

^b^ Correlation (*r*) significance values are shown as *p* <0.05 (*∗*) and <0.01 (*∗∗*).

## Supplementary Materials

Supplementary Materials Supp. Table S1. The phenotypic variation of the nondestructive traits and the above-ground biomass and the final seed yield across the four stress treatments drought (D), heat (H), and combined heat and drought (H+D) compared to the control (C) after different days of treatments (DAT). The average phenotypic performance of 12 Brassica genotypes is shown on the left and their response (%) to D, H, and H+D compared to C (stress-control/control) is shown italicised in brackets. Supp. Figure S1. Diurnal temperatures in the controlled environment growth cabinets during the 7-d temperature treatment period. The lights were switched on at 06:00 h and switched off at 22:00 h. Supp. Figure S2. Average soil water content over time in each treatment. 0% SWC is oven-dried soil. Supp. Figure S3. Plant growth imaging with LemaTec Scanalyser after treatment of drought and/or heat stress. A. The imaging system utilizes two cameras and produces a top view, 0° side view, and a 90° rotated side view in each image capture. B. The images for each plant are processed using the Scanalyser imaging software grid automated algorithm analysis. The pot, tags, etc. are removed by simple thresholding of the image, and thus only the pixels from plant tissues are segmented. The two-dimensional plant area for each image in pixels is then calibrated and converted to mm^3^ to create a plant volume.
