# Supplementary material for: Nondestructive Phenomic Tools for the Prediction of Heat and Drought Tolerance at Anthesis in Brassica Species
Source: Plant Phenomics. 2019 May 22;2019:3264872. doi: 10.34133/2019/3264872 (PMC7718632; doi:10.34133/2019/3264872)

Supp. Table S1. The phenotypic variation of the non-destructive traits and the above-ground biomass and the final seed yield across the four stress treatments drought (D), heat (H) and combined heat and drought (H+D) compared to the control (C) after different days of treatments (DAT). The average phenotypic performance of 12 Brassica genotypes is shown on the left and their response (%) to D, H and H+D compared to C (stress-control/control) is shown italicised in brackets.

^a^ The phenomic traits include (i) six in-cabinet rapid phenotyping traits: photochemical reflectance index (PRI), photosystem II quantum yield (Qy), leaf conductance (LC), and the temperature differences between (a) bud and ambient environment (T1), (b) leaf and ambient environment (T2), and (c) bud and leaf (T3); (ii) three photosynthetic traits derived from A/Ci curves: maximum carboxylation rate allowed by Rubisco (Vc*max*), photosynthetic electron transport rate (ETR) and the rate of triose phosphate use (TPU); and (iii) two plant growth imaging derived phenotyping traits: whole plant volume (VolWP) and flower volume (VolF).

^b^ The agronomic traits include fresh weight (FW) of the whole plant at DAT7, and seed yield (SY) and 100-seed weight (SW) of each plant at maturity.

Supp. Fig. S1 Diurnal temperatures in the controlled environment growth cabinets during the 7-d temperature treatment period. The lights were switched on at 06:00 h and switched off at 22:00 h.


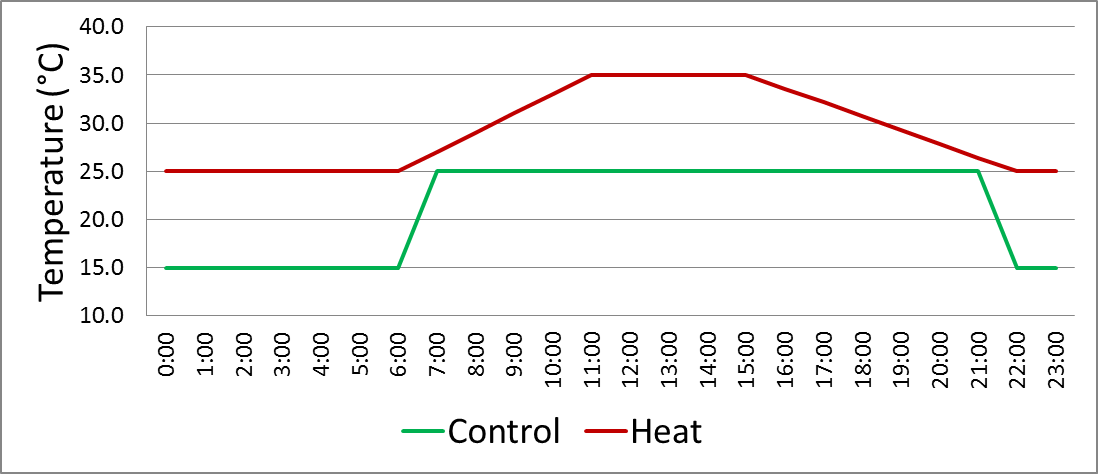


Supp. Fig. S2: Average soil water content over time in each treatment. 0% SWC is oven-dried soil.
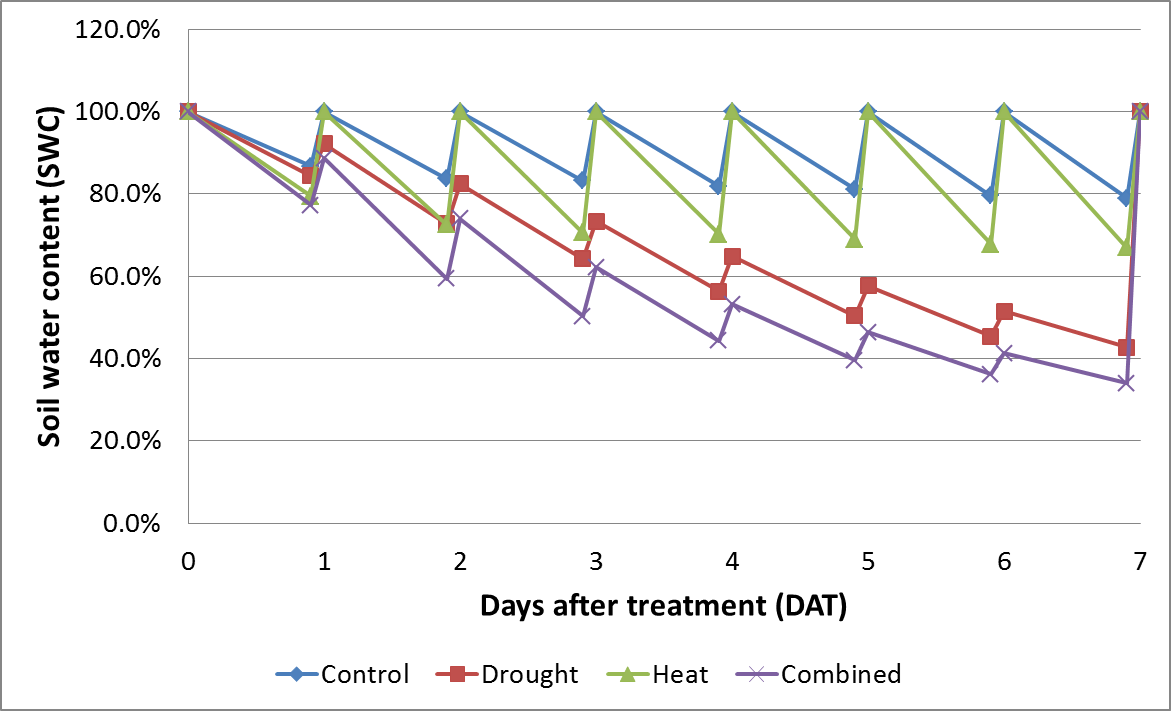


Supp. Fig. S3: Plant growth imaging with LemaTec Scanalyser after treatment of drought and/or heat stress. A. The imaging system utilizes two cameras and produces a top view, 0˚ side view, and a 90˚ rotated side view in each image capture. B. The images for each plant are processed using the Scanalyser imaging software grid automated algorithm analysis. The pot, tags etc are removed by simple thresholding of the image, and thus only the pixels from plant tissues are segmented. The two-dimensional plant area for each image in pixels is then calibrated and converted to mm^3^ to create a plant volume.


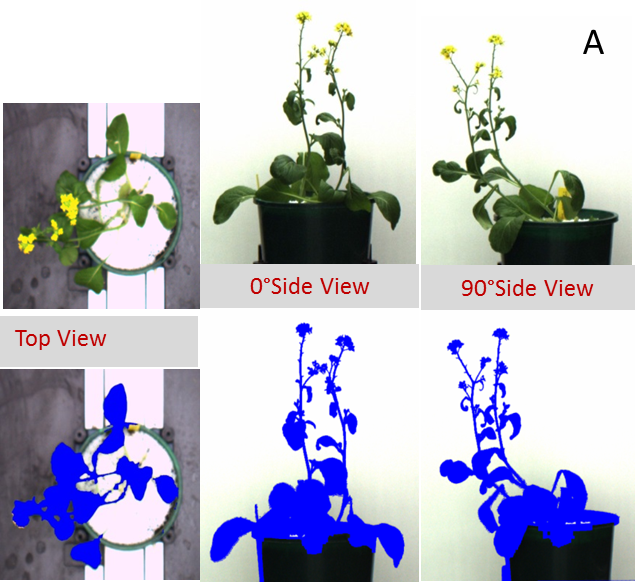


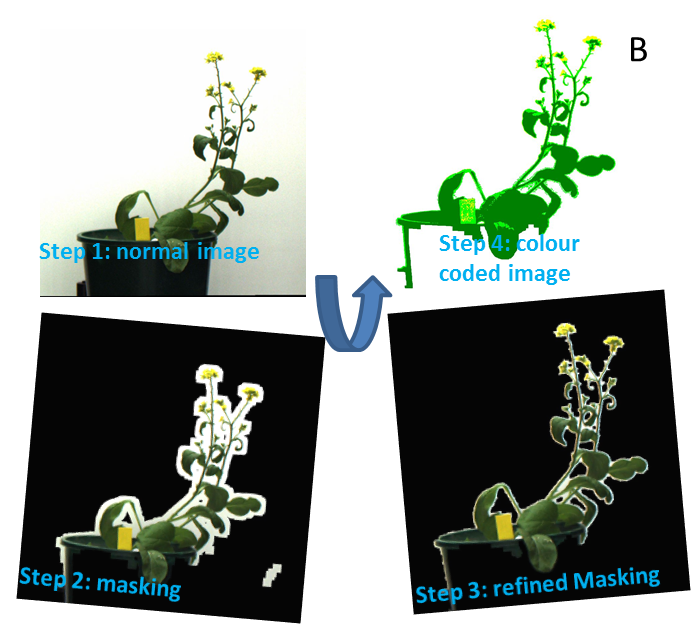

Supplement: Supplementary Materials — Supp. Table S1. The phenotypic variation of the nondestructive traits and the above-ground biomass and the final seed yield across the four stress treatments drought (D), heat (H), and combined heat and drought (H+D) compared to the control (C) after different days of treatments (DAT). The average phenotypic performance of 12 Brassica genotypes is shown on the left and their response (%) to D, H, and H+D compared to C (stress-control/control) is shown italicised in brackets. Supp. Figure S1. Diurnal temperatures in the controlled environment growth cabinets during the 7-d temperature treatment period. The lights were switched on at 06:00 h and switched off at 22:00 h. Supp. Figure S2. Average soil water content over time in each treatment. 0% SWC is oven-dried soil. Supp. Figure S3. Plant growth imaging with LemaTec Scanalyser after treatment of drought and/or heat stress. A. The imaging system utilizes two cameras and produces a top view, 0° side view, and a 90° rotated side view in each image capture. B. The images for each plant are processed using the Scanalyser imaging software grid automated algorithm analysis. The pot, tags, etc. are removed by simple thresholding of the image, and thus only the pixels from plant tissues are segmented. The two-dimensional plant area for each image in pixels is then calibrated and converted to mm3 to create a plant volume. [file 3264872.f1.docx]
